# Using routine health care data to develop and validate a system dynamics simulation model of frailty trajectories in an ageing population

**DOI:** 10.1080/20476965.2025.2459364

**Published:** 2025-01-31

**Authors:** Tracey England, Bronagh Walsh, Sally Brailsford, Carole Fogg, Simon de Lusignan, Simon DS Fraser, Paul Roderick, Scott Harris, Abigail Barkham, Harnish P Patel, Andrew Clegg

**Affiliations:** a School of Health Sciences, University of Southampton, Southampton, UK; b Southampton Business School, University of Southampton, Southampton, UK; c Nuffield Department of Primary Care Health Sciences, University of Oxford, Oxford, UK; d School of Primary Care, Population Sciences, and Medical Education, Faculty of Medicine, University of Southampton, Southampton, UK; e Southern Health NHS Foundation Trust, Southampton, UK; f Southampton General Hospital, University Hospital Southampton NHS Trust, Southampton, UK; g NIHR Southampton Biomedical Research Centre, Southampton Centre for Biomedical Research, Southampton General Hospital, Southampton, UK; h Academic Unit for Ageing & Stroke Research, University of Leeds, Bradford Teaching Hospitals NHS Foundation Trust, Bradford, UK

**Keywords:** Frailty, system dynamics, ageing population, transitions, validation

## Abstract

Frailty is common in older adults and has a substantial impact on patient outcomes and service use. Information to support service planning, including prevalence in middle-aged adults and patterns of frailty progression at population level, is scarce. This paper presents a system dynamics model describing the dynamics of frailty and ageing within a population of patients aged ≥50, based on linked data for 2.2 million patients from primary care practices in England. The purpose of the model is to estimate the incidence and prevalence of frailty in an ageing population over time. The model was developed in consultation with stakeholders (patients, carers, clinicians, and commissioners) and validated against another large dataset (1.38 million patients) from Wales. It was then scaled up to the population of England, using Office for National Statistics projections (to 2027). The baseline results, subject to the assumption that the frailty transition parameters remain constant over this period, suggest that the number of people living with frailty will increase as the population ages, and that those with mild-moderate frailty are likely to have the greatest impact on demand. This paper focuses on model development and validation, highlighting the benefits and challenges of using large routine health datasets.

## 1. Introduction

Frailty is a long-term condition where an individual is vulnerable to internal and external stressors (e.g., infection and falls) and is associated with poor health outcomes and high service use (Clegg et al., 2013). Informally, frailty can be described as a loss of resilience resulting from a cumulative impact of chronic conditions and ageing) that prevents people from “bouncing back” quickly after an illness, an accident, or some other stressful event (Age UK, 2024). Frailty may be identified following a physical assessment (Fried et al., 2001) or by using a frailty score or “index”, based on an accumulation of conditions or disabilities, which can be derived from routinely collected data using a validated measure such as the electronic frailty index (eFI) (Aguayo et al., 2017; Clegg et al., 2016) which is used in clinical practice across England (National Health Service [NHS], 2023a), and Scotland using the Scottish Primary Care Information Resource (SPIRE) (Health Improvement Scotland, 2023). Frailty screening and intervention are now a requirement of the GP contract in England (NHS, 2019) and the use of the eFI is expanding throughout primary care (Clegg et al., 2016), making this an appropriate measure for studying frailty in the population. Recognising that a patient has a degree of frailty can prompt primary care professionals to review the care offered and to tailor it to that person’s needs.

Frailty is strongly associated with age, with prevalence estimates varying from 4% to 50%, depending on the measure of frailty and population studied (Siriwardhana et al., 2018) with a pooled prevalence of 24% (measured using a frailty index) reported across 62 countries (O’Caoimh et al., 2020). For example, with the electronic frailty index (eFI), 50% of people (931,541 registered from over 500 UK primary care practices) aged 65–95 have some level of frailty (Clegg et al., 2016). In a recent study, amongst a large cohort of over 2 million patients aged ≥50 in England, frailty prevalence increased from 26.5% to 38.9% over a 12-year period between 2006 and 2017 (Walsh et al., 2023).

Although frailty research has expanded over the last decade, there has been limited simulation modelling of frailty progression over time, particularly in the middle-aged and young-old age groups of a population. Models have tended to represent the physiology of frailty (Lipsitz, 2008) or how the body’s stimulus-response mechanism may change following a stressor event (Varadhan et al., 2008) rather than consider the population level dynamics of the condition and the service needs of the patients. The need for better planning and modelling of demand within the primary care system is well recognised (British Geriatrics Society and the Royal College of General Practitioners, 2015) and has been reiterated in a recent Health Foundation report (Watt et al., 2022) which highlights the implications for health and social care as well as for other public services from an ageing population. Howlett et al. (2021) comment on the need for computational models that can improve our understanding of how frailty is related to age, changes in frailty states and mortality. Farrell et al. (2021) suggest that computational models for ageing and frailty need to be systems level models, preferably stochastic (to capture variability), predictive, and parameterised from data from large populations of ageing individuals.

### 1.1. Previous work

System dynamics (SD) has frequently been used to model healthcare systems (Cassidy et al., 2019; Hirsch & Homer, 2004; Lane et al., 2000; Royston et al., 1999), particularly when a more high-level, aggregated, strategic view is needed (Homer & Hirsch, 2006). In its quantitative (stock-flow) form, SD is a powerful tool that can be used to represent different levels of illness in the population of interest (Cassidy et al., 2019; Husain et al., 2021), where transitions between stages can be affected by disease progression or healthcare interventions (Darabi & Hosseinichimeh, 2020; Davahli et al., 2020; Graham et al., 2023). It is ideally suited to model large populations where individual variability is not a major consideration. A stochastic approach may be better for small subgroups, or where it is necessary to follow individual people’s trajectories over time, but this is not an issue here. However, in a recent literature review (Williams et al., 2021), of 62 journal articles referenced as Operational Research or Management Science models related to care planning for frail and elderly patients, only three specifically mentioned SD. Two of the three focused on how to improve inpatient length of stay (LoS) and reduce the problem of delayed hospital discharge (Rashwan et al., 2015; Walker & Haslett, 2001) whilst the third focused on dementia (Cepoiu-Martin & Bischak, 2018); none of the three considered frailty.

Studies related to dementia can provide useful learning for simulation of frailty, due to similarities in patient age and disease progression (Thompson et al., 2012, 2014). A recent UK study used a hybrid simulation approach in which SD was used to represent the population-level effects, and agent-based simulation for the patient-level characteristics, to model dementia in the over 65 population (Evenden et al., 2021). Both dementia studies consider progression/transition to more severe states of illness and include mechanisms to age the patients in the population. Both promote the usefulness of SD in looking at population-level outcome measures and care planning under different “what-if” scenarios.

To our knowledge, there is an evidence gap in the literature concerning studies that use SD modelling with data from large longitudinal studies as an approach to understanding frailty at the population level. To date, only one report that we have found uses SD in relation to frailty and was conducted by West Kent Local Care, in association with Whole Systems Partnerships, and concerned the care needed for patients with frailty (defined as a patient having six or more disabilities and roughly equivalent to moderate/severe frailty in our study) and complex needs in Kent and Medway, UK (Whole System Partnership, 2018). The model considered the effect of different interventions such as multidisciplinary team (MDT) working in GP clusters and pre-hospital urgent care to avoid hospital admissions and reduce length of stay. The model considered people aged 60+ in 5-year age bands and focused on specific aspects of the healthcare system.

### 1.2. Aim of this paper

Given findings of a prevalence of frailty of nearly 11% in people aged 50–64 and its impact on transitions to more severe frailty states (Walsh et al., 2023), there is therefore a need to develop a model which can be used to examine frailty within an ageing population. This paper describes the development and validation of an SD stock-flow model representing frailty onset and progression in people aged ≥50 years, stratified by age. The purpose of the model was to estimate the incidence and prevalence of frailty in an ageing population over time. Further research will consider the associated health care demand and workforce planning needed for health and social care provision for people living with frailty. With the recent publication of the NHS workforce plan (National Health Service NHS, 2023b), the model developed in this study may prove useful in providing clinicians and planners with some of the missing pieces of the jigsaw (Palmer & Rosen, 2023) such as an estimate of the number of people living with frailty that need health and social care services in the next 10–15 years and the associated workforce and training needs. The development of this model was part of a larger study entitled Frailty Dynamics (National Institute for Health and Care Research (NIHR) Health and Social Care Delivery Research (HSDR) 16_116_43) whose aim was to describe the dynamics of frailty in the older adult population, and its impact on patients and health care services, to inform the commissioning and service development of future services (Fogg et al., 2022). This wider study provided data on the epidemiology of frailty (Walsh et al., 2023), obtained through analysis of a large, routine primary care dataset, provided by the Royal College of General Practitioners (RCGP) Research and Surveillance Centre (RSC). This dataset was used to estimate the original model parameters. Extensive stakeholder engagement exploring patient/carer and professional experience also informed the model structure and the choice of experimental scenarios.

The remainder of this paper is structured as follows. Section 2 (Materials and Methods) describes the model, the data, and the processes of stakeholder engagement, verification, and validation. Section 3 presents the results from running the England-wide model for 2006–27, while Section 4 discusses the implications of these results, the limitations of the model, the benefits, and challenges of using large routine health datasets for modelling, and ongoing future research.

## 2. Materials and methods

### 2.1. The system dynamics model

The model is a quantitative, data-driven stock-flow model representing flows of people between different age/frailty compartments. The transition rates between compartments were derived from the datasets described in the Data section below.

The study population was divided into four age groups, reflecting the groupings reported in literature relating to older adults’ healthcare service provision: 50–64, 65–74, 75–84, and ≥85 (Fogg et al., 2022; Walsh et al., 2023). This approach, which follows Evenden et al. (2021), assumes that the average age in each age group remains unchanged in each timestep, and hence the age-related mortality and frailty transition rates apply to the whole stock. This relatively minor assumption avoids the need for more complicated methods described in the literature (Eberlein & Thompson, 2013; Sterman, 2000) to deal the so-called blending problem, i.e., within-stock differences in flow rates. Each age group was further divided according to the frailty state measured by the electronic frailty index (eFI) (Clegg et al., 2016). The eFI is a cumulative deficit score comprising 36 deficits across disease states, symptoms/signs, abnormal laboratory values, and indicators of disability. An eFI score (calculated as the number of deficits present in an individual as a proportion of the total possible on the 1^st^ January each year) was provided for each participant in the dataset (Fogg et al., 2022; Walsh et al., 2023). The eFI was categorised into frailty states as: Fit (0–0.12), Mild (0.13–0.24), Moderate (0.25–0.36), or Severe (>0.36). Hence the modelled population contained 16 subgroups.

**Figure 1.**
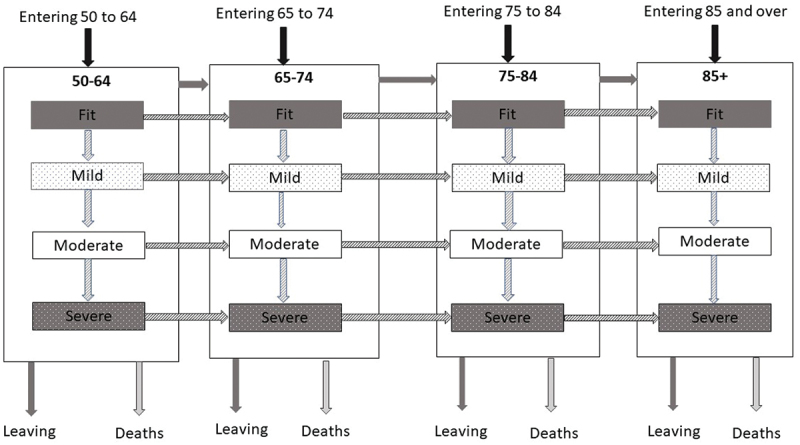
Schematic showing the 16 age/frailty subgroups and movement between the groups.

A schematic of the underlying model structure is shown in Figure 1, which was developed in consultation with all stakeholders. The state transitions agreed with stakeholders were ageing, moving from one frailty state to another, and dying. However, since the RSC dataset at the time only represented 8% of all GP practices in England (Correa et al., 2016), new patients could join the cohort, while others could “de-register” by leaving an RSC network-registered GP practice for one that was not included in the dataset. The numbers of new joiners and de-registrations were, therefore, significant and hence these inflows and outflows (respectively labelled “Entering” and “Leaving” in Figure 1) had to be included in the model.

In the full model, there are three stocks for each of the 16 age/frailty combinations, representing patients who are alive, have died, or have left the cohort, within that particular age group and frailty state. The model therefore consists of 48 stocks (i.e., numbers of people in the different age/frailty states) and 72 flows (i.e., movement of people as they transition from one state to another over time). Given the inconsistency in clinical evidence of the reversibility of frailty through interventions, the main model assumption that frailty is not reversible (i.e., patients cannot move to an improved frailty state) greatly simplified the modelling and was agreed after discussion with the clinical stakeholders. Given this lack of certainty, the simplifying assumption of no “backwards” transitions was included.

The model was developed in AnyLogic (version 8.7.3). Several of the flows are functions of transition proportions that are assumed to remain constant over time, whereas others are time dependent, reflecting how the dynamics of a particular subgroup change over time. The exact forms of the flow equations were determined by examining the proportion of patients that transitioned to the next category in a given time period (during the RCGP RSC cohort study period (2006–2017)) and using multiple least squares regression to include the relevant covariates (e.g., time, time^2^, time^3^). A detailed explanation of the model equations is included in the Technical Appendix, along with a Table presenting all the model parameters.

### 2.2. Stakeholder engagement

We were keen to engage a range of stakeholders throughout the whole process as other studies have acknowledged that failure to involve stakeholders throughout a simulation model’s development can lead to the findings not being implemented (Kotiadis & Tako, 2021). In the wider Frailty Dynamics study, both patients and carers as well as commissioners and providers of healthcare services to older people living with frailty were recruited to three stakeholder events (with 61 professionals and 8 members of the public invited and approximately 50% attending). We were conscious that these groups may not have had prior experience of simulation modelling, so we were careful to tailor the sessions accordingly. As these events took place during the COVID-19 pandemic, a mixture of in-person and virtual events were offered. The findings from the data analysis were discussed, as well as the model itself. These were not group model-building sessions as normally understood in SD, and no causal loop diagram was developed. Rather, people were asked about the model assumptions (as noted above); whether anything important had been omitted from the model; and whether the proposed model outputs were useful. In the final event, clinicians and commissioners were asked to comment on the results and whether they felt the model provided a realistic representation of frailty in the population. Other issues important to these particular stakeholders were whether the results remained stable when the model was run into the future, and whether it was feasible to adjust the model parameters to reflect potential future changes in service provision.

Several “What-if” scenarios were also discussed with both stakeholder groups. The clinicians and commissioners were keen to test the effect of hypothetical interventions to reduce or delay the onset of frailty, and also reduce the rate of progression through the frailty states. In this paper, we present the development and validation of the simulation model using the baseline parameters, i.e., assuming that current clinical practice does not change and demographic trends continue. Scenario analyses are reported elsewhere (Walsh et al., 2024).

### 2.3. Data

The RCGP RSC dataset represents a nationally representative cohort of primary care patients from GP practices in England, held at Oxford University (Correa et al., 2016). The cohort consisted, at the time of the study, of 1,107,481 people registered at 419 GP practices at the start of the data period (January 2006), rising to 2,177,656 at the end of December 2017. The RSC data informed the model structure and parameter estimates, including the initial stock levels in each of the 16 population subgroups and all the flow rates (Walsh et al., 2023).

The dataset used for external model validation was extracted from the Secure Anonymised Information Linkage (SAIL) databank (Databank, 2021; Ford et al., 2009; Jones et al., 2019; Lyons et al., 2009). It included patients aged ≥50 registered with GPs in Wales, at any time between 2006 and 2017 inclusive. The dataset represents approximately 80% of the whole population of Wales (Swansea University, 2021) since some people are not registered with a GP and others might be registered with a GP in England. Following exclusions, the final dataset included 1,380,959 patients contributing 11,090,653 follow-up years. As the Welsh population is broadly similar to that of England, the SAIL dataset was a useful comparison for the purposes of validation of the SD model. There are, however, some differences in demographic and socio-economic structure that were reflected in the cohort datasets (Table 1) and needed to be considered in the model development and validation process.

**Table 1.** Initial percentage of patients in each of the 16 frailty/age subgroups in each cohort.

Cohort & Age Group	Fit (%)	Mild (%)	Moderate (%)	Severe (%)	Percentage of cohort
RCGP RSC					
50–64	46.8	5.14	0.49	0.048	52.5
65–74	16.95	6.27	1.08	0.13	24.4
75–84	7.82	6.51	2.14	0.45	16.9
85+	1.93	2.53	1.30	0.42	6.2
Percentage of cohort	73.5	20.5	5.0	1.1	100
SAIL					
50–64	43.16	8.65	0.94	0.08	52.8
65–74	14.83	8.15	1.66	0.21	24.9
75–84	6.49	7.02	2.54	0.54	16.6
85+	1.49	2.45	1.36	0.42	5.7
Percentage of cohort	66.0	26.3	6.5	1.3	100

The initial proportion of patients in each of the 16 subgroups in each cohort at the start of 2006 are given in Table 1. Previous analyses of the RSC cohort found that even in the youngest age group, substantial numbers are already living with frailty (Walsh et al., 2023). The same was true in the SAIL cohort, although prevalence at ages 50–64 was even higher in this population; roughly 11% (62,731 out of 579,199) of the RCGP RSC cohort had some degree of frailty in 2006, whilst in SAIL the proportion was 18% (81,013 out of 442,480). Table 1 shows that in terms of age structure, the two cohorts were similar at the start of 2006, but there were some differences in the prevalence of frailty; a higher proportion were recorded as frail in the SAIL cohort (RCGP: 26.5% cf. SAIL: 34%). The median eFI score for the RSC cohort is lower (0.056 (0.028–0.111)) compared with that of SAIL (0.083 (0.028–0.139)), suggesting a fitter population.

In terms of socio-economic status, comparison of deprivation quintiles using the Welsh Index of Multiple Deprivation (Welsh Index of Multiple Deprivation, 2011) for SAIL, and the 2015 version of the Index of Multiple Deprivation for RCGP RSC, where 1 represents the most deprived and 5 the least, suggests the SAIL cohort has higher levels of deprivation, with 37.8% in the two most deprived categories, versus 26.8% for RCGP RSC. Abel et al. (2016) describe similar higher levels of deprivation in Wales compared with England in their comparison of UK constituent countries indices of multiple deprivation. We concluded, therefore, that the observed differences in the prevalence of frailty were more likely to be due to deprivation than age structure (which is very similar in Table 1) and could easily be adjusted for in the model by scaling the frailty prevalence in SAIL to match the RCGP RSC values. The scaling factors applied to account for socio-economic differences between the two populations are given in the Technical Appendix (Table A3). With these adjustments, the two datasets/populations were considered to be sufficiently similar to enable us to use SAIL as a validation dataset for the model.

### 2.4. Verification and validation

Verification and validation are known to be a challenge in SD modelling (McLucas et al., 2012) with (Sterman, 2000) suggesting that validation of SD models is actually impossible. The aim of verification and validation is to build confidence in the model, determining whether it is useful and fit for purpose, often using a mixture of qualitative and quantitative tests throughout model development. The larger and more complex the model, the more difficult verification and validation can become. The standard statistical approach is to compare the model output with historical data not used to develop the model, but this does not guarantee that the model structure and parameters are all correct; a good match to observed data may just be a lucky coincidence. A major contribution of this study is that we have had access to two very large primary care population datasets over the same time period to develop and validate the SD model.

As part of the verification, the model was run for the same time period (2006–2017) as the data source in the RCGP RSC cohort study, using the same initial population conditions (Fogg et al., 2022). The model estimated the number of patients in each stock, and the number of state transitions, were then compared against the observed data at the start of each year. Time plots were used for graphical comparison of the observed and estimated data. Error statistics, using the mean absolute percentage error (MAPE), were also used to assess agreement between the observed data and model output over the 12 years of data.

Table 2 shows the MAPEs for each of the stocks and flows associated with the 16 population subgroups. The MAPE (with each variable equally weighted) for the model was 7.06%, indicating that the model provides a close approximation to the (RCGP RSC) patient cohort data. As one would expect, the smaller subgroups had larger errors (e.g., moderate, and severe in the two younger age groups). Comparing the error statistics in the final two columns of Table 2 (for Frailty Transitions and Ageing), the errors are slightly larger (suggesting a poorer fit to the data) for the fit to mild frailty transitions, with the model tending to underestimate the number of these transitions. One possible explanation is (as noted earlier) the eFI was only calculated once each year in the RSC dataset, thus potentially missing the exact date when a patient’s eFI score reached the threshold between fit and mildly frail. In reality, this particular transition may be difficult to observe; moreover, in general fit people visit their GP less frequently than people with mild or moderate frailty. However, as there were some areas where accuracy was lower, further examination was warranted in the external validation process using the SAIL data.

**Table 2.** Verification (Internal validation) using the RCGP RSC cohort: MAPE^1^ for each age/frailty subgroup.

	Alive	Entering the cohort	Dying	Deregistration	Frailty Transitions	Ageing
50–64						
Fit	0.45	1.96	5.35	5.77		10.35
Mild	1.42	2.37	6.55	6.93		11.53
Moderate	6.93	5.57	7.58	7.94		14.91
Severe	7.77	15.84	4.00	10.22		19.45
Fit to Mild					24.04	
Mild to Moderate					3.43	
Moderate to Severe					4.83	
65–74						
Fit	3.89	5.43	5.64	4.93		8.03
Mild	2.91	6.18	7.39	7.24		6.99
Moderate	3.44	7.15	5.75	9.90		11.82
Severe	5.39	22.48	7.40	9.45		9.55
Fit to Mild					9.30	
Mild to Moderate					7.83	
Moderate to Severe					10.49	
75–84						
Fit	3.75	4.39	8.16	1.83		10.94
Mild	1.53	5.42	6.30	5.58		9.07
Moderate	3.50	4.43	5.91	6.52		4.35
Severe	3.99	11.02	4.47	7.62		8.54
Fit to Mild					14.46	
Mild to Moderate					5.50	
Moderate to Severe					6.54	
85+						
Fit	4.70	4.67	5.49	6.12		n/a
Mild	2.43	4.01	3.22	4.71		n/a
Moderate	2.23	5.52	2.51	8.38		n/a
Severe	10.44	16.08	5.26	11.87		n/a
Fit to Mild					6.23	
Mild to Moderate					6.79	
Moderate to Severe					5.29	

For the external validation, the starting age/frailty stocks were populated with their equivalents from the 2006 SAIL cohort. The SAIL data had greater population coverage than RCGP RSC (80% versus approximately 8%) and therefore lower entry and de-registration rates than were used in the original model, which meant that these had to be modified to reflect those rates in the external validation. Initially, the RCGP RSC entry and de-registration values were multiplied by constant scaling factors, so they matched the rates within SAIL; see Table A3 in the Technical Appendix.

The model outputs (number of patients living, deaths, leaving GP practices, ageing, and transitioning to the next frailty state) were compared with the observed data from the SAIL cohort. Table 3 shows the MAPEs for each age/frailty category. The error statistics associated with the number of living patients range in size from 1.38% (Moderate, 75 to 84-year-olds) to 11.05% (Severe, 65 to 74-year-olds) with almost all less than 10% and nine of the 16 error statistics less than 5%. The MAPEs are similar to those in the internal validation exercise for most of the age groups with the 75 to 85-year-old group experiencing lower MAPE values in most of the frailty categories.

**Table 3.** Validation using the SAIL cohort: MAPE (%) for each age/frailty subgroup.

	Living	Entering the cohort	Dying	Deregistration	Frailty Transitions	Ageing
50–64						
Fit	2.09	5.05	9.48	4.77		8.17
Mild	1.41	5.64	8.36	7.38		12.3
Moderate	6.45	8.35	5.37	11.73		10.26
Severe	6.73	18.86	5.12	10.92		21.38
Fit to Mild					3.05	
Mild to Moderate					4.94	
Moderate to Severe					3.85	
65–74						
Fit	2.4	16.99	9.69	10.95		6.08
Mild	7.10	25.24	9.84	7.26		9.21
Moderate	5.58	28.12	4.01	18.2		2.78
Severe	11.05	48.84	1.13	15.36		14.05
Fit to Mild					6.68	
Mild to Moderate					8.03	
Moderate to Severe					3.33	
75–84						
Fit	3.31	19.97	11.2	4.13		10.12
Mild	4.32	21.98	10.7	6.6		3.24
Moderate	1.38	23.23	6.62	12.34		7.39
Severe	2.33	27.49	1.99	4.32		3.19
Fit to Mild					10.61	
Mild to Moderate					4.39	
Moderate to Severe					5.37	
85+						
Fit	9.12	12.02	8.01	7.38		n/a
Mild	4.15	15.76	6.70	8.23		n/a
Moderate	2.93	19.19	3.09	11.13		n/a
Severe	8.92	34.64	4.43	4.3		n/a
Fit to Mild					4.45	
Mild to Moderate					5.97	
Moderate to Severe					7.38	

The MAPEs associated with deaths were lower for some of the moderately and severely frail groups, and in most of the 16 age/frailty subgroups were no more than 4% worse than the original model. For the frailty transitions, the MAPEs were within acceptable levels and ranged between 3.05% (Fit to Mild in the 50 to 64-year-old age group) and 10.61% (Fit to Mild in the 75 to 84-year-old age group). Improvements compared with the RCGP RSC model were also observed in the MAPEs associated with ageing.

Although the MAPEs for simulation outputs were acceptable, the MAPEs associated with the number of patients entering the SAIL cohort varied between 5.05% and 48.84%; the fit was worse in all 16 population subgroups, compared with the RCGP RSC. The error statistics for de-registrations were also worse, by as much as 9%. With both these flows, the larger errors were due in part to the smaller proportions of people entering the SAIL cohort (other than by reaching the age of 50) and leaving it (other than by dying) compared with the RCGP RSC cohort, which had much lower population coverage than SAIL. Applying the de-registration rates from the RCGP data to the SAIL cohort may have also over-estimated the number of de-registrations resulting in increased error statistics. SAIL entry flow data in 2008 and 2016 were initially thought to be due to additional data and/or GP practices being uploaded into the SAIL database, but on further examination these appeared to be due to wider demographic trends, related to “baby boom” periods between the Second World War and the 1960s, a phenomenon which also needed to be taken into account in the development of the national simulation model for England. This suggested that merely scaling the RCGP RSC entry and deregistration rates was not an ideal approach. However, since the aim was to extend the model to cover the entire population of England (ignoring migration), so that people would only enter the model by reaching age 50 and would only leave by dying, errors due to new registrations and de-registrations were not a concern as these particular flows do not occur in the whole population.

The overall error statistic for external validation, weighting all variables of interest equally was 9.53%. This was still within acceptable bounds, approximately 2.5% worse than with the original model. After adjusting for the demographic and socio-economic differences between the two cohorts, external validation shows that the underlying model structure is robust and can be used for different populations, although this may require appropriate adjustment of the input parameters to account for socio-economic and demographic differences that impact on population health status. The fact that sample data were used to estimate the original state transitions may have led to some of the model parameters being over-fitted to that particular sample, rather than taking account of wider demographic trends, an issue that needs to be considered when using large-scale data for model building.

The final step was to scale up the model to provide a national view of frailty progression in England, and to run the model with an extended time horizon of 10 years. The model was populated with Office for National Statistics (ONS) 2021 mid-year population estimates and principal projections for England (Office for National Statistics, 2022a, 2022b) for 2006–2027. As noted earlier, both entries and de-registrations were removed from this final model, under the assumption of a negligible net migration effect at the national level (Office for National Statistics, 2022a). Hence, once a patient reaches age 50, they will remain in the model until they die. This national model therefore took account of broader population trends in the study period, compared with the original model based on the RCGP RSC data alone. This reduced the likelihood of over-fitting to sample data, albeit a very large sample.

## 3. Results

**Figure 2.**
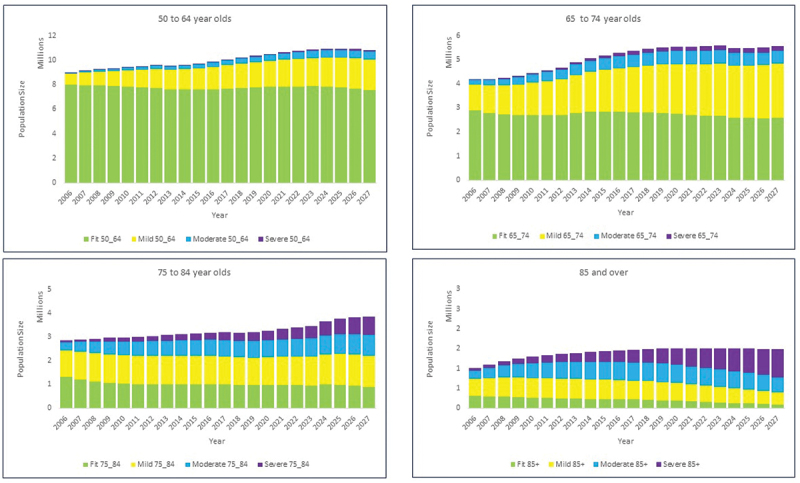
Baseline projected estimates of the 16 age/frailty population subgroups, England, 2006–2027.

The baseline model was run from 2006 to 2027 with the assumption that after 2017, health care service delivery as well as individual lifestyles remain constant thereby mitigating any changes on the onset or progression of frailty. We recognise this assumption may not be correct, especially as it ignores the impact of the COVID-19 pandemic on older people, and therefore the results should be interpreted with caution. Figure 2 shows the numbers of people in each age group, broken down by frailty state, for each year from 2006 to 2027. The model estimates that the ≥50 population increases from 17.02 million in 2006 to 20.05 million in 2017 and will reach 21.75 million in 2027. Those experiencing some level of frailty (mild, moderate, or severe) is estimated to increase from 4.48 million in 2006 to 8.32 million in 2017 and to 10.59 million in 2027. Considering those experiencing moderate or severe frailty, the model estimates that the number will increase from 1.02 million in 2006 to 2.91 million in 2017 and to 4.28 million in 2027.

Table 4 shows the changes between 2017 and 2027 (both in absolute numbers and as a percentage of the total population aged over 50) in each age/frailty subgroup. Over the period 2017 to 2027, the model suggests that the percentage of people aged ≥50 experiencing some level of frailty will rise from 41.5% in 2017 to 48.7% in 2027. The total number of people categorised as Fit falls by 4.7% while the number with mild frailty increases by 16.1%. The most striking change, however, is for people with moderate and severe frailty: the total number in these two categories increases by 47% from 2.9 million to 4.3 million, comprising almost 20% of the population aged ≥50. This means that compared with 2017, the model estimates that by 2027 an additional 2.27 million people (with 1.37 million living with moderate or severe frailty) may require some level of care (as increasing frailty is associated with increased care needs or service use (Fried et al., 2001; Ge et al., 2020; Ikonen et al., 2022)). This would be a significant increase in a relatively short period of time.

**Table 4.** Percentage of the total population aged over 50, and numbers of people, in each age/frailty subgroup in 2017 and in 2027.

	2017		2027	
Age/frailty group	%	Number	%	Number
Fit				
50–64	38.36	7,689,306	34.85	7,580,985
65–74	14.04	2,813,696	11.92	2,593,440
75–84	4.99	1,000,342	4.15	902,502
85+	1.09	218,329	0.42	92,415
Mild				
50–64	9.43	1,890,524	11.31	2,460,389
65–74	9.34	1,871,634	10.35	2,252,318
75–84	5.87	1,175,918	5.95	1,294,121
85+	2.38	476,438	1.39	302,014
Moderate				
50–64	1.93	387,793	3.02	657,204
65–74	2.60	521,885	2.40	521,665
75–84	3.42	686,230	4.02	875,014
85+	2.3	460,649	1.77	384,709
Severe				
50–64	0.32	63,186	0.64	139,944
65–74	0.74	148,237	0.96	208,367
75–84	1.61	323,287	3.61	785,707
85+	1.59	318,311	3.24	704,303
Total		20,046,000		21,755,000

Unsurprisingly it is the older age groups who experience the highest levels of frailty, but it may be surprising to some readers to learn that the prevalence of frailty is still relatively high in the youngest age group, and because this age group contains so many people it contributes significantly towards overall need and demand for services. The results indicate that the 50–64 age group will form 28.1% of the total frail population in 2017, rising to 30.7% in 2027. The model suggests that by 2027 there will be around 3.25 million people aged 50–64 living with frailty. In particular, it can be seen from Table 4 that severe frailty more than doubles in this age group between 2017 and 2027, and moderate frailty increases by 50%. The older age groups, while smaller in terms of absolute numbers, will see a similar doubling in the proportion with severe frailty, although the proportions with moderate frailty do not increase so sharply (and even, in the 85+ age group, decrease slightly).

## 4. Discussion

We have developed and validated an SD model representing frailty onset and progression in people aged ≥50 years, stratified by age, using large-scale, routine health service data. We have used the model to estimate the incidence and prevalence of frailty states over time. Our baseline results suggest that there could be a significant increase in demand for both health and social care associated with frailty. Whilst clinical attention has focused on severe frailty and the oldest old, the main growth could be in mild/moderate frailty in the middle aged and younger old groups that are not generally considered in planning. These groups may require medication reviews or falls risk assessments (if appropriate). Future work will consider the associated healthcare demand, workforce and other resources needed for health and social care provision for people living with frailty. A recent report by the Health Foundation contained projections suggesting that in 2040 there would be an extra 2.5 million adults above 20 years of age in England living with a major illness (defined using the Cambridge Multimorbidity Score (CMS)) compared with the situation in 2019 (Watt et al., 2022). As the Health Foundation report states, there is no silver bullet to reduce the growth in the number of people living with major illness/multi-comorbidities (Watt et al., 2022); the same can be said for those living with frailty. Clearly, the substantive findings from our study are highly relevant for policy and decision-makers involved in planning future services for people living with frailty. The approach outlined here provides the potential to quantify service requirements (staff and facilities) and how they might change over time especially when combined with population projections. This work also provides useful learning in relation to the use of large-scale, routine health service data for development and validation of simulation models in this field.

### 4.1. Limitations

A strength of this study is that we have demonstrated that we were able to use real-world, nationally representative, large-scale data to develop and validate a model that generates useful and stable projections over a 10-year time period. However, despite the large size of the dataset, there were challenges in fitting some model parameters that became apparent during the external validation process. These included the issues with entries and de-registrations from the cohort sample (flows which would be negligible in the population as a whole), the impact of socio-economic differences and wider demographic trends such as the “baby boomers”, as described earlier. Arguably, if we had been using a smaller dataset, we might have been more alert to these issues, as it would have been more obvious that our data flows were not necessarily representative of the flows in the wider population. A key learning point is that even although the RCGP RSC cohort had been shown to be nationally representative in terms of cross-sectional demographic characteristics (Correa et al., 2016), it was still necessary to take account of wider demographic trends when extending the model to the entire population of England. Due to these differences in population flows in our primary datasets, questions about the representativeness of the RCGP RSC cohort on which transition probabilities are based, and therefore the generalisability of the model to the wider English population, could arise. However, by using the SAIL dataset in the external validation we have been able to examine the behaviour patterns in a cohort which has a much higher coverage of a national population before adapting the model and using ONS population principal projections to describe the age structure and ageing process of people aged ≥50 in England. A similar approach of using ONS population projections with prevalence estimates (based on linked primary care data) for 20 conditions was used in a recent Health Foundation study on comorbidities to ensure changes in the underlying ageing population (such as the Baby boomers) were captured ahead of model projections for England up to 2040 (Watt et al., 2022). The external validation in this study process has showed that whilst the model structure is universally generic and could be transferable to other populations or countries, work would be needed to identify suitable data sources to adapt the model for different regions, countries, and time periods.

As with all models, some simplifications were necessary due to the complexity and limitations of the data on which the model was developed. First, the eFI was updated annually (on 1^st^ January) based on GP data from the preceding 12 months; we did not attempt to model transitions based on the actual dates of transitions between frailty states. In reality, frailty progression is a gradual process, and it may be difficult to specify precisely when some of the deficits that constitute the eFI actually began (as opposed to when they were noted in a patient’s record). There was no evidence to suggest that this annual update would lead to bias (in either direction) but as noted earlier, it may have had a small effect on the error statistics for some transitions since age was updated on a more granular basis. Second, the model was developed with age and frailty as the main variables of interest. While gender, deprivation, and ethnicity have also been shown to be associated with frailty (Majid et al., 2020; Romero-Ortuno et al., 2014; Stow et al., 2022) and (Walsh et al., 2023), the marginal benefits of including one or more of these factors explicitly in the model would be outweighed by the exponential increase in complexity. The “curse of dimensionality” would make the model almost impossible to parameterise, and hence far less useful in practice. In the model, the frailty transition rates are instead adjusted for these important covariates, based on analysis of the RCGP RSC cohort (Walsh et al., 2023). While this is a strength, there is also a limitation in using a single composite measure of transition probability, especially as stakeholders commented that they would like to be able to understand differences in the future needs of their populations in relation to underlying factors such as deprivation which may become more important in the face of austerity measures and the current cost of living crisis. It is, however, possible to address these issues by adjusting the model’s input parameters (e.g., transition and mortality rates) to reflect different population structures, as we did for the SAIL population. Third, the model assumes that frailty is not reversible. Analysis of the RSC dataset showed that, although frailty scores did occasionally improve over time, this was uncommon. The extent to which such improvements are an artefact of the scoring system used to measure frailty is unclear. Moreover, implementation issues can mean that changes in the eFI deficit metrics are not captured consistently in patient records. In 3.9% of patients, reversals such as from Moderate to Mild were an artefact of the scoring of polypharmacy (i.e., being prescribed multiple medications) (Walsh et al., 2023) and could not be assumed to be due to the true underlying frailty state of the patient. Fourthly, as noted in the Materials and Methods section, like many ageing chain models in the literature the model is subject to the blending problem, especially for the widest age group (50–64). While our approach clearly involves some loss of accuracy, we decided that the use of methods like continuous cohorting (Eberlein & Thompson, 2013) was not justified given the uncertainty around other model parameters and the fact that other factors such as migration are not included. An intermediate approach of using 1-year age bands would require a major reparameterization exercise (over 500 parameters) using the RCGP RSC dataset. Finally, the assumption that frailty transition rates, service provision, and other model parameters remain constant after 2017 is clearly a limitation; in particular, it ignores the impact of the COVID-19 pandemic. Future work, which considers workforce issues, will address this.

## 5. Conclusions

Involving stakeholder groups throughout the study has ensured that the simulation is informed by, and responds to, the real-world context, whilst at the same time allowing us to communicate findings arising from the data analysis and model development to patients, carers, and clinicians. Consultation has provided valuable opportunities to gather information and feedback from different groups in relation to the simulation structure and aims, data needs and sources, and the scenarios of interest. We also obtained valuable feedback on how the findings from the study should be communicated to commissioners and those planning services to older people living with frailty. This will inform preparation of guidance which includes simulation scenarios that address different socio-demographic contexts as well as modifications to health services.

COVID-19 had an impact on the timing and delivery of planned stakeholder events. We were, however, able to adapt our approach to hold a mix of face-to-face and virtual events with patient and service providers as restrictions permitted, during which we were able to concentrate on stages 3 (Specify the conceptual model) to 5 (Experiment with model) of the PartiSim Framework (Tako & Kotiadis, 2015) designed to support facilitated simulation modelling in healthcare.

Having a population level model based on a large routine healthcare dataset will enable us to understand the current and future primary and secondary care needs and the associated costs of older people (aged ≥50) living with frailty (Fogg et al., 2023). Scenario analysis will allow examination of both preventative and management strategies to address the potential increase in the number of people living with frailty due to an ageing population. Further scenario analyses could consider the effect of austerity and the COVID-19 pandemic on frailty incidence and prevalence in the national population aged 50 and over. Ongoing research will build on the simulation presented here to explore the workforce requirements in primary, secondary and social care to support the over 50s population as they become older and frail. Developing a demand-based workforce model may prove useful in relation to the NHS’ workforce plan for the next 15 years (National Health Service NHS, 2023b).

## Supplementary Material

Supplementary_Material_170424.docx

## Data Availability

The data used in the study are secondary data and are retained by the data providers (RCGP RSC and SAIL). The data are not publicly available as restrictions apply to the availability of these data, which were used following approvals and data sharing agreements for the current study.

## Note

1. MAPE = mean absolute percentage error.
