# Supplementary material for: Using routine health care data to develop and validate a system dynamics simulation model of frailty trajectories in an ageing population
Source: Health Syst (Basingstoke). 2025 Jan 31;14(3):195–207. doi: 10.1080/20476965.2025.2459364 (PMC12364089; doi:10.1080/20476965.2025.2459364)
Supplement: Supplementary_Material_170424.docx [file THSS_A_2459364_SM7277.docx]

Using system dynamics and routine health care data to develop and validate a simulation model of frailty trajectories in an ageing population.

Contents

[Technical Appendix 2](#_Toc159576963)

[Underlying structure of the Frailty Dynamics system dynamics (SD) Population model 2](#_Toc159576964)

[Difference Equations 2](#_Toc159576965)

[External Validation 8](#_Toc159576966)

[Example output from the system dynamics population model developed in AnyLogic (8.7.3). 9](#_Toc159576967)

# Technical Appendix

The technical appendix provides more detail on the underlying structure of the system dynamics model: in particular, the difference equations used to represent the stocks and flows within the model and the scaling adjustments carried out during the external validation.

## Underlying structure of the Frailty Dynamics system dynamics (SD) Population model

### Difference Equations

The equations included in the Population Model are used to estimate the number of patients in certain categories at a certain point after the start of January 2006. The 6 categories we are interested in are:

- The number of patients in each of the age/frailty groups who are alive at a given time;
- The monthly number of patients who change frailty status (remaining in the same age group), assuming worsening of frailty, captured by the eFI score: i.e., Fit to Mild, Mild to Moderate, Moderate to Severe;
- The monthly number of patients who age and move into the next age group, assuming they stay in the same frailty category;
- The monthly number of new patients joining each of the age/frailty groups;
- The number of patients who die each month;
- The number of patients who deregister or are lost to follow-up each month.

Solving difference equations related to these 6 categories provides estimates of the number of patients in each age/frailty group over time (the stocks and flows within the model).

As the over 50’s population is divided into 4 age bands (50-64, 65-74, 75-84 and 85+) and each age band has 4 measures of frailty according to the patients’ eFI scores (Fit, Mild, Moderate and Severe), there are 16 population subgroups in total.

The population model based on the Royal College of General Practitioners (RCGP) Research and Surveillance Centre (RSC) cohort data initially considers 12 years from 1^st^ January 2006 through to the end of December 2017, with a monthly time step.  The projections based on the population model and scaled up to the national level consider an extra 10 years, from the end of December 2017 through to the end of December 2027.

As the equations within the model cannot be typically solved analytically, we use a numerical approximation algorithm. This involves starting with an initial number of patients in each of the 16 age/frailty groups and then adding or subtracting a given number of patients each month (Equation 1). The number of patients that are added or removed depends on a group of equations (currently 80 for the Population Model, due to the 16 population subgroups).

The typical structure of the equation capturing the **monthly** change in each age/frailty population subgroup (${population}_{j}$) is as follows, with the variables described in Table A.1, illustrated by an example where ${population}_{j}$ is people aged 65-74 with Mild frailty:

*Equation 1*

$$\frac{\Delta\left( {population}_{j} \right)}{dt}={entryflow}_{j}+{frailtytransitionflow}_{j-1 to j}-{frailtytransitionflow}_{j to j+1}-{ageingflow}_{j}+{ageingflow}_{j-1}-{deaths}_{j}-{deregistrations}_{j}$$

Table A1: Description of the variables in Equation 1

| Variable | Description | Example for Mild(65-74) |
| --- | --- | --- |
| $\boldsymbol{entryflow}_{\boldsymbol{j}}$ | Number of new patients who join age/frailty subgroup *j* each month |  |
| $\boldsymbol{frailtytransitionflow}_{\boldsymbol{j-1 to j}}$ | Monthly number of patients who transition from the previous frailty category, but stay the same age | From Fit(65-74) to Mild(65-74) |
| $\boldsymbol{frailtytransitionflow}_{\boldsymbol{j to j+1}}$ | Monthly number of patients who transition to the next frailty category, but stay the same age | From Mild(65-74) to Moderate(65-74) |
| $\boldsymbol{ageingflow}_{\boldsymbol{j}}$ | Monthly number of patients who transition to the next age band but stay in the same frailty category | From Mild(65-74) to Mild(75-59) |
| $\boldsymbol{ageingflow}_{\boldsymbol{j-1}}$ | Monthly number of patients who transition from the previous age group, but stay in the same frailty category | From Mild(50-64) to Mild(65-74) |
| $\boldsymbol{deaths}_{\boldsymbol{j}}$ | Monthly number of deaths in age/frailty subgroup *j* | From Mild(65-74) to Dead(65-74) |
| $\boldsymbol{deregistrations}_{\boldsymbol{j}}$ | Monthly number of patients in age/frailty subgroup *j* who have de-registered from a RCGP RSC GP practice or are lost to follow-up | From Mild(65-74) to Deregistered(65-74) |

Equations 2 to 4, representing the flow rates (entry, ageing and frailty transition) were derived by multiple least squares regression with powers of time $t$ as covariates. The parameters $a,b,c, d$and $e$ are the coefficients of $t, t^{2}, t^{3}$ and (where applicable) $t^{4}$ in the regression equations; they take different values for each flow type and each combination of age group and frailty class.  The numerical values of these parameters are given in Table A2.

The expressions for **entry flow** (${entryflow}_{j}$) are typically of the form given in Equation 2:

*Equation 2*

${entryflow}_{j}=\frac{\left( a+bt+ct^{2}+dt^{3}+et^{4} \right)}{12}$ where $t$ is the number of months since the start of January 2006

The expressions for the **frailty transition flows** (e.g., ${frailtytransitionflow}_{j-1 to j}$ ) are typically of the form given in Equation 3:

*Equation 3*

$${frailtytransitionflow}_{j-1 to j}={population}_{j}*\frac{\left( a+bt+ct^{2}+dt^{3} \right)}{12}$$

The expressions for the **ageing flows** (e.g., ${ageingflow}_{j}$) are typically of the form given in Equation 4:

*Equation 4*

$${ageingflow}_{j}={population}_{j}*\frac{\left( a+bt+ct^{2}+dt^{3}+et^{4} \right)}{12}$$

The expressions for the **exit flows due to death** (${deaths}_{j}$) are typically of the form given in Equation 5 where ${propdie}_{j}$ is the proportion of the population subgroup that die in a given month.

*Equation 5*

$${deaths}_{j}={population}_{j}*{propdie}_{j}$$

The expressions for the exit flows due to deregistration from RCGP RSC GP or loss to follow-up

(e.g., ${deregistrations}_{j}$) are typically of the form given in Equation 6 where ${propdereg}_{j}$ is the proportion of the population subgroup that deregister in a given month.

*Equation 6*

$${deregistrations}_{j}={population}_{j}*{propdereg}_{j}$$

Table A2: Parameter values (RCGP RSC cohort)

|  | Entry | Ageing | Deaths  ($\boldsymbol{propdie}_{\boldsymbol{j}}$) | Deregistrations  ($\boldsymbol{propdereg}_{\boldsymbol{j}}\boldsymbol{)}$ | Frailty |
| --- | --- | --- | --- | --- | --- |
| 50 – 64  *Fit* | a: 6.7 *10^4^  b: -5.38 *10^2^  c: 11.76  d: -5.47 *10^-2^ | a: 4.64 * 10^-2^  b: 4.1 * 10^-5^  c: 4.7 * 10^-7^  d: -1.6 * 10^-10^  e: 4.8 * 10^-14^ | 1.06 * 10^-4^ | 2.47 * 10^-3^ |  |
| *Mild* | a: 4.19 * 10^3^  b: -18  c: 1.01  d: -4.39*10^-3^ | a: 8.18 * 10^-2^  b: -7.5 * 10^-5^  c: 3.47 * 10^-7^  d: 3.47 * 10^-10^  e: -9.65 * 10^-14^ | 2.9 * 10^-4^ | 2.3 * 10^-3^ |  |
| *Moderate* | a: 2.50 * 10^2^  b: 1.41  c: 4.97 * 10^-2^  d: -1.13*10^-4^ | a: 8.3 * 10^-2^  b: 5.1*10^-5^  c: -2.78 *10^-8^  d: 1.74 *10^-11^  e: 9.65 *10^-15^ | 5.5 * 10^-4^ | 3.0 * 10^-3^ |  |
| *Severe* | a: 16.73  b: 4.24 *10^-1^  c: -4.2 *10^-4^  d: 3.89 *10^-5^ | a: 7.7 * 10^-2^  b: 1.7 *10^-4^  c: -6.94 *10^-8^  d: 5.79 *10^-12^  e: 4.82 * 10^-15^ | (8.3* 10^-4^) + (5 * 10^-9^t^2^) | 4.1 * 10^-3^ |  |
| *Fit to Mild* |  |  |  |  | a: 2.4 * 10^-2^  b: 1.0 *10^-6^  c: -6.95 *10^-10^  d: |
| *Mild to Moderate* |  |  |  |  | a: 3.47 * 10^-2^  b: -5.83 *10^-6^  c: $5.56*{10}^{-9}$  d: -5.21 * 10^-13^ |
| *Moderate to Severe* |  |  |  |  | a: 3.6 * 10^-2^  b: -2.5 *10^-5^  c: 3.47 *10^-8^  d: -1.16 *10^-11^ |
|  |  |  |  |  |  |
| 65 – 74  *Fit* | a: 8.82 * 10^3^  b: -1.23 * 10^2^  c: 7.07 * 10^-1^  d: 1.93 * 10^-3^  e: -1.34*10^-4^ | a: 6.9 * 10^-2^  b: -2.53 *10^-5^  c: 2.5 *10^-9^  d:  e: | 2.85 * 10^-4^ | 2.17 * 10^-3^ |  |
| *Mild* | a: 2.37 * 10^3^  b: -5.05 * 10^2^  c: 1.01  d: -4.66 * 10^-3^ | a: 9.0 * 10^-2^  b: 1.58 * 10^-6^  c: -5.0*10^-7^  d:  e: | 5.6 * 10^-4^ | 2.3 * 10^-3^ |  |
| *Moderate* | a: 2.63 * 10^2^  b: -2.71  c: 6.25 *10^-2^  d: -1.50 *10^-4^ | a: 9.97 * 10^-2^  b: 5.0 *10^-5^  c: -2.78 *10^-8^  d:  e: | 9.8 * 10^-4^ | 3.8 * 10^-3^ |  |
| *Severe* | a:  b: 1.67  c: -3.2 * 10^-2^  d: 2.18 *10^-4^ | a: 8.99 * 10^-2^  b: 1.22 *10^-3^  c: -7.4 *10^-6^  d:  e: | (1.67 * 10^-3^)+ (1 * 10^-8^ t^2^) | 6.2 * 10^-3^ |  |
| *Fit to Mild* |  |  |  |  | a: 8.08 * 10^-2^  b: -1.67 *10^-5^  c: 6.2 *10^-9^  d: |
| *Mild to Moderate* |  |  |  |  | a: 7.18 * 10^-2^  b: -2.0 *10^-4^  c: 1.4 *10^-8^  d: |
| *Moderate to Severe* |  |  |  |  | a: 6.15 * 10^-2^  b: -2.5 *10^-5^  c: 1.39 *10^-8^  d: |
|  |  |  |  |  |  |
| 75 – 84  *Fit* | a: 4.32 * 10^3^  b: -9.1 * 10  c: 1.42  d: -6.12 *10^-3^ | a: 6.09 * 10^-2^  b: -2.9 *10^-4^  c: 2.0 *10^-6^  d:  e: | 8.18 * 10^-4^ | 3.1 * 10^-3^ |  |
| *Mild* | a: 2.90 * 10^3^  b: -5.47 * 10  c: 1.04  d: -4.98*10^-3^ | a: 7.54 * 10^-2^  b: -3.0 *10^-4^  c: 1.0 *10^-6^  d:  e: | 1.27 * 10^-3^ | 3.6 * 10^-3^ |  |
| *Moderate* | a: 6.20 * 10^2^  b: -7.43  c: 0.20  d: -8.72 *10^-4^ | a: 8.02 * 10^-2^  b: 6.0 *10^-5^  c: -1.0 *10^-9^  d:  e: | 1.75 * 10^-3^ | 2.6 * 10^-3^ |  |
| *Severe* | a: 2.71 * 10  b: 2.89  c: -4.50 *10^-2^  d: 3.31 * 10^-4^ | a: 7.75 * 10^-2^  b: 4.0 *10^-4^  c: -2.0 *10^-6^  d:  e: | (2.6 * 10^-3^) + (6 * 10^-8^t^2^) | 9.28 * 10^-3^ |  |
| *Fit to Mild* |  |  |  |  | a: 2.06 * 10^-1^  b: -1.4 * 10^-3^  c: 5.9 * 10^-6^  d: |
| *Mild to Moderate* |  |  |  |  | a: 1.15 * 10^-2^  b: -7.1 * 10^-4^  c: 5.0 * 10^-5^/12  d: |
| *Moderate to Severe* |  |  |  |  | a: 1.03 * 10^-1^  b: -5.0 *10^-4^  c: 4.0 *10^-6^  d: |
|  |  |  |  |  |  |
| 85+  *Fit* | a: 1.85 * 10^3^  b: -3.29 * 10  c: 4.95 * 10^-1^  d: -2.08*10^-3^ |  | 3.1 * 10^-3^ | 7.65 * 10^-3^ |  |
| *Mild* | a: 1.78 * 10^3^  b: -1.66 * 10  c: 4.67 * 10^-1^  d: -2.68*10^-3^ |  | 3.7 * 10^-3^ | 8.3 * 10^-3^ |  |
| *Moderate* | a: 6.03 * 10^2^  b: -3.38  c: 2.03 * 10^-1^  d: -1.07 *10^-3^ |  | 3.7 * 10^-3^ | 9.8 * 10^-3^ |  |
| *Severe* | a: 1.87 * 10  b: 6.78  c: -1.13 * 10^-1^  d: 8.43 *10^-4^ |  | (3.7 * 10^-3^) + (2*10^-7^t^2^) | 1.41 * 10^-2^ |  |
| *Fit to Mild* |  |  |  |  | a: 2.95 * 10^-1^  b: -1.8 *10^-3^  c: 1.3 *10^-5^  d: |
| *Mild to Moderate* |  |  |  |  | a: 2.10 * 10^-1^  b: -1.1 *10^-3^  c: 8.0 *10^-6^  d: |
| *Moderate to Severe* |  |  |  |  | a: 1.38 * 10^-1^  b: -8.0 *10^-4^  c: 7.0 *10^-6^  d: |

### External Validation

Table A3: Model Parameterisation and Adjustment Factors applied during the External Validation

| Frailty category and age group | Entry into cohort | Death | De-registrations | Ageing into next age group | Frailty Transition |
| --- | --- | --- | --- | --- | --- |
| Fit | | | | | |
| 50-64 | 0.504 | 1.208 | 0.411 | No change | 1.8 |
| 65-74 | 0.279 | 1.159 | 0.503 | 0.90 | Adjusted parametric form for Fit to Mild transition: 0.1134 – 0.0003t + 0.0000008t^2^ |
| 75-84 | 0.210 | 1.130 | 0.494 | 0.895 | No change |
| 85+ | 0.155 | 1.069 | 0.64 |  | Adjusted parametric form for Fit to Mild transition:  0.2907-0.0011t +0.000003t^2^ |
| Mild | | | | | |
| 50-64 | 1.100 | 0.835 | 0.288 | 1.16 | 1.1 |
| 65-74 | 0.399 | 0.921 | 0.353 | No change | No change |
| 75-84 | 0.277 | 0.953 | 0.412 | No change | No change |
| 85+ | 0.200 | 0.94 | 0.407 |  | Adjusted parametric form for Mild to Moderate transition: 0.1936 + 0.0003t – 0.000003t^2^ |
| Moderate | | | | | |
| 50-64 | 1.368 | 0.947 | 0.362 | 1.389 | 1.17 |
| 65-74 | 0.570 | 0.944 | 0.469 | 1.15 | No change |
| 75-84 | 0.356 | 0.879 | 0.396 | 0.853 | No change |
| 85+ | 0.273 | 0.987 | 0.389 |  | No change |
| Severe | | | | | |
| 50-64 | 1.370 | 1.062 | 0.370 | 1.701 |  |
| 65-74 | 0.95 | 1.000 | 0.468 | 1.23 |  |
| 75-84 | 0.592 | 0.994 | 0.381 | No change |  |
| 85+ | 0.491 | 0.993 | 0.434 |  |  |

### Example output from the system dynamics population model developed in AnyLogic (8.7.3).

Figure A1 depicts the graphical model output (from the AnyLogic software) and shows the number of people living with frailty.


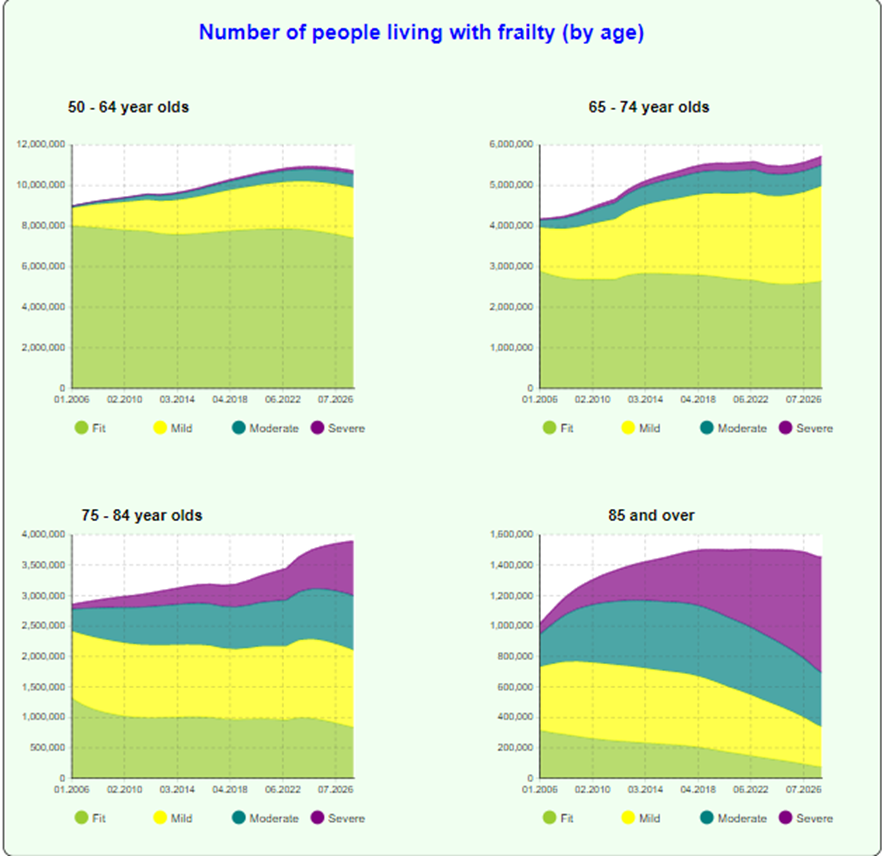


Figure A1 Example output from SD population model
